# Supplementary material for: Degradation-Suppressed Cocoonase for Investigating the Propeptide-Mediated Activation Mechanism
Source: Molecules. 2022 Nov 20;27(22):8063. doi: 10.3390/molecules27228063 (PMC9693254; doi:10.3390/molecules27228063)
Supplement: Supplementary file 1 [file molecules-27-08063-s001.zip › molecules-2025642-supplementary.pdf]

Supplementary Materials

Table S1. Primer sequences used in this study.

Primer name	Primer direction	Primer sequence	Mutation
pMT1	forward	5′ ATCTCAGGTGTGACCGTTCGCATT 3′	Lys63Gly
pMT1	reverse	5′ GGTCACACCTGAGATCCCTTCAAT 3′	Lys63Gly
pMT2	forward	5′ CCGGATGGTACCGCACTGCTGGTTAGTGGA 3′	Lys131Gly, Lys133Ala
pMT2	reverse	5′ CAGCAGTGCGGTACCATCCGGTACAGACGA 3′	Lys131Gly, Lys133Ala
pAO4	forward	5′ GCTGCTGCTTGCATTGAAGGGATC 3′	His56Ala
pAO4	reverse	5′ AATGCAAGCAGCAGCAGTGAGAAT 3′	His56Ala
pAO5	forward	5′ AACAATGCTTTTGCGATTGTCACG 3′	Asp99Ala
pAO5	reverse	5′ CGCAAAAGCATTGTTCTTCGTTTT 3′	Asp99Ala
pAO6	forward	5′ GGCGATGCTGGTGGTCCTGCCGTG 3′	Ser193Ala
pAO6	reverse	5′ ACCACCAGCATCGCCTTGACACGA 3′	Ser193Ala
pAO7	forward	5′ CTGTCAGACGACGATGACAAGATTGTCGGTGGGGAA 3′	Lys8Asp, Glu10Asp, Glu11Asp
pAO7	reverse	5′ AATCTTGTCATCGTCGTCTGACAGCGCTTCAGAATC 3′	Lys8Asp, Glu10Asp, Glu11Asp
pAO9	forward	5′ CTGTCAGACGACGATGACAAGATTGTCGGTGGGGAA 3′	Lys8Asp, Glu10Asp, Glu11Asp
pAO9	reverse	5′ AATCTTGTCATCGTCGTCTGACAGCGCTTCAGAATC 3′	Lys8Asp, Glu10Asp, Glu11Asp
pAO10	forward	5′ CTGTCAGACGACGATGACAAGATTGTCGGTGGGGAA 3′	Lys8Asp, Glu10Asp, Glu11Asp
pAO10	reverse	5′ AATCTTGTCATCGTCGTCTGACAGCGCTTCAGAATC 3′	Lys8Asp, Glu10Asp, Glu11Asp
pNS1	forward	5′ CTGTCAGACGACGAAGAGAAGATTGTC 3′	Lys8Asp
pNS1	reverse	5′ TTCGTCGTCTGACAGCGCTTCAGAAT 3′	Lys8Asp
T_7	forward	5′ TAATACGACTCACTATAGG 3′	-
T_7	reverse	5′ CCCAAGGGGTTATGCTA 3′	-

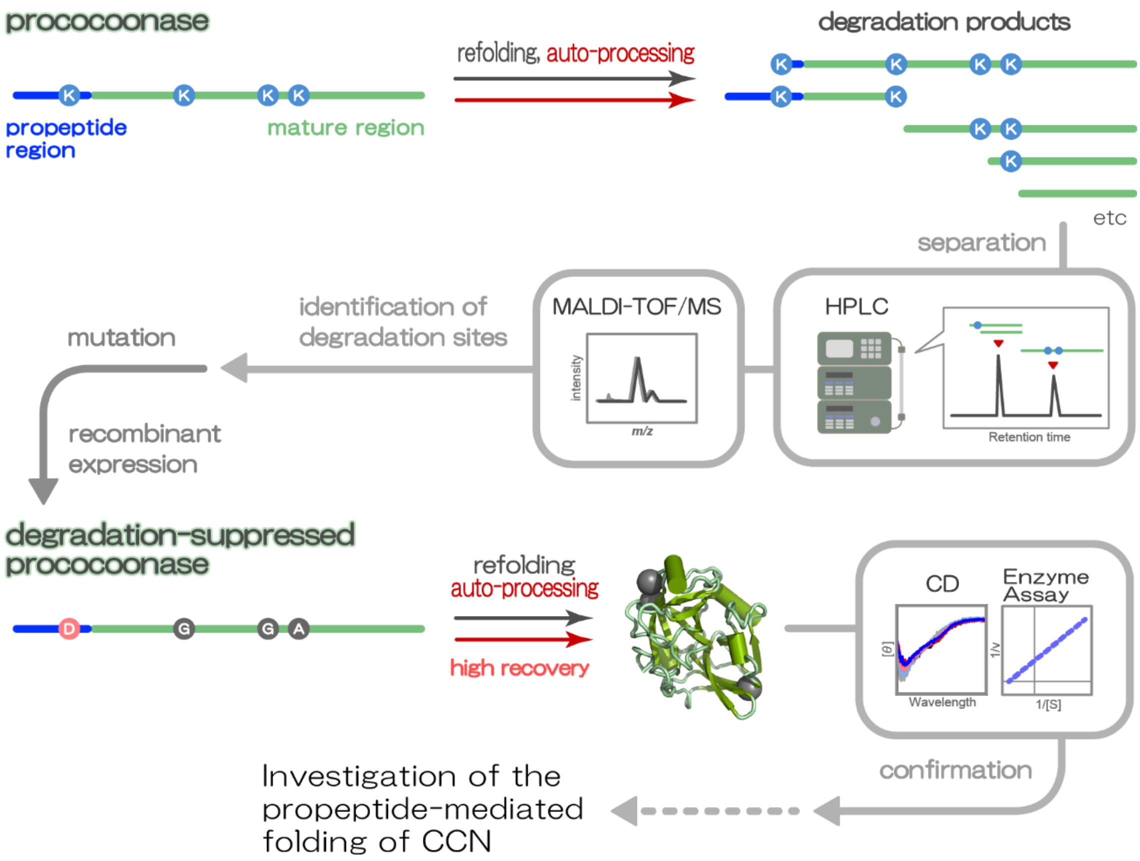

Supplemental Figure S1. Schematic diagram showing the preparation of the degradation-suppressed prococoonase.

```
human trypsin  1 MNPLLILTFV AAALAAPFDD DDKIVGGYNC EENSVPYQVS 40
cocoonase                   1 TDSEALSKD EEKIVGGEEI SINKVPYQAY 29

41 LN--S-G-YH FCGGSLINEQ WVVSAGHCYK S--RIQVRLG EHNIEVLEGN 84
30 LLLQKDNEYF QCGGSIISKR HILTAAHCIE GISKVTVRIG SSNSN---KG 76

85 EQFINAAKII RHPQYDRKTL NNDIMLIKLS SRAVIN-ARV STISLPTAP- 132
77 GTVYTAKSKV AHPKYNSKTK NNDFAIVTVN KDMAIDGKTT KIITLAKEGS 126

133 -PATGTKCLI SGWGNTASSG ADYPDELQCL DAPVLSQAKC EASYPGKITS 181
127 SVPDKTKLLV SGWGATSEG- GSSSTTLRAV HVQAHSDDEC KKYFR-SLTS 174

182 NMFCVGFLEG GKDSCQGDSG GPVVCNGQLQ GVVSWGDGCA QKNKPGVYTK 231
175 NMFCAGPPEG GKDSCQGDSG GPAVKGNVQL GVVSFGVGCA RKNNPGIYAK 224

232 VYNYVKWIKN TIAANS
225 VSAAAKWIKS TAGL
```

Supplemental Figure S2. Homology alignment of procoonase and human trypsinogen.

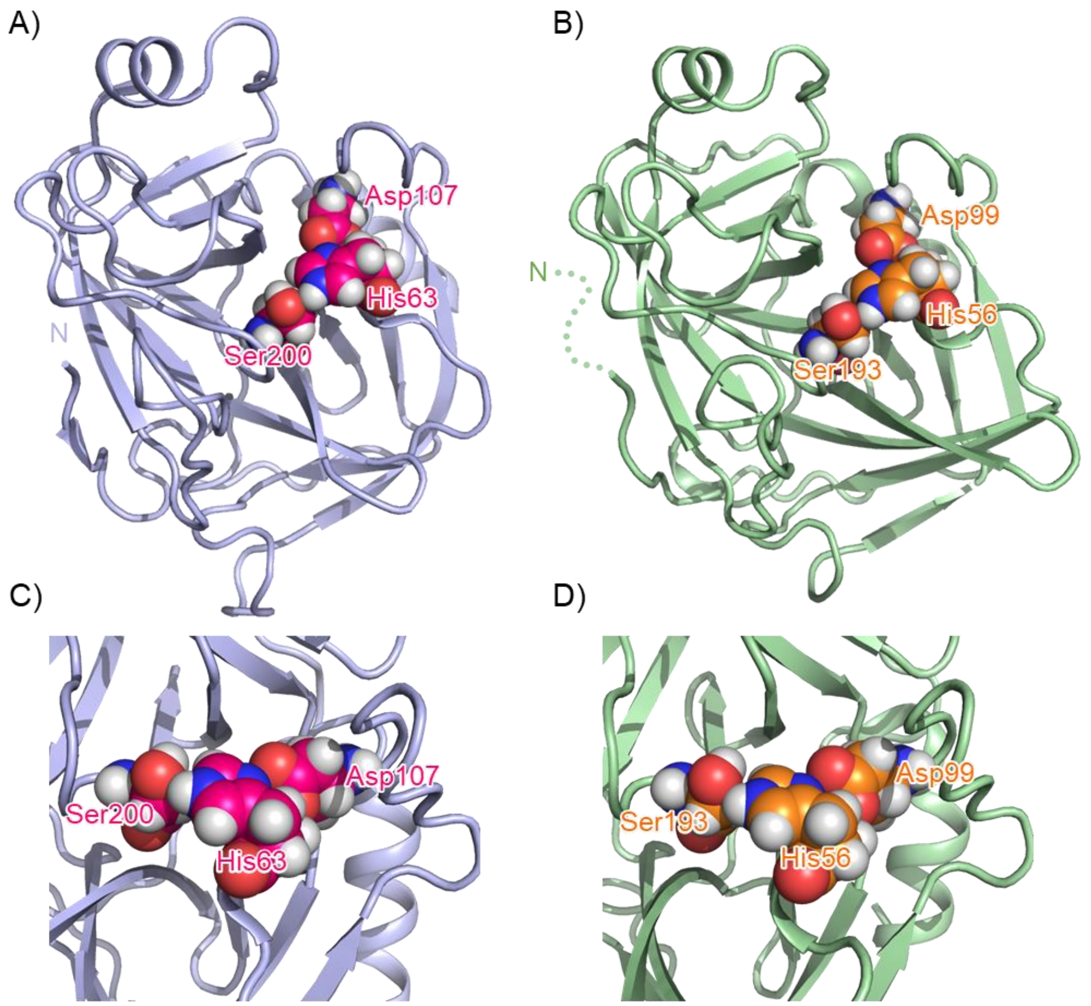

Supplemental Figure S3. Molecular models of trypsin (A, C; PDB ID 5T3H) and cocoonase (B, D).

The catalytic residues are indicated by space filling atoms using the PyMol software.

```
                 9          18          27          36          45          54
5'  ACG GAT TCT GAA GCG CTG TCA AAA GAC GAA GAG AAG ATT GTC GGT GGG GAA GAA
    --- --- --- --- --- --- --- --- --- --- --- --- --- --- --- --- --- ---
    Thr Asp Ser Glu Ala Leu Ser Lys Asp Glu Glu Lys Ile Val Gly Gly Glu Glu

                63          72          81          90          99         108
    ATC TCC ATC AAC AAA GTA CCC TAT CAG GCC TAT CTT CTA CTG CAG AAA GAC AAC
    --- --- --- --- --- --- --- --- --- --- --- --- --- --- --- --- --- ---
    Ile Ser Ile Asn Lys Val Pro Tyr Gln Ala Tyr Leu Leu Leu Gln Lys Asp Asn

               117         126         135         144         153         162
    GAG TAC TTC CAG TGT GGC GGT TCC ATC ATC TCC AAA CGT CAC ATT CTC ACT GCT
    --- --- --- --- --- --- --- --- --- --- --- --- --- --- --- --- --- ---
    Glu Tyr Phe Gln Cys Gly Gly Ser Ile Ile Ser Lys Arg His Ile Leu Thr Ala

               171         180         189         198         207         216
    GCT CAT TGC ATT GAA GGG ATC TCA AAA GTG ACC GTT CGC ATT GGT AGC AGT AAT
    --- --- --- --- --- --- --- --- --- --- --- --- --- --- --- --- --- ---
    Ala His Cys Ile Glu Gly Ile Ser Lys Val Thr Val Arg Ile Gly Ser Ser Asn

               225         234         243         252         261         270
    TCG AAT AAA GGC GGC ACA GTG TAT ACT GCG AAA AGC AAG GTT GCC CAT CCG AAA
    --- --- --- --- --- --- --- --- --- --- --- --- --- --- --- --- --- ---
    Ser Asn Lys Gly Gly Thr Val Tyr Thr Ala Lys Ser Lys Val Ala His Pro Lys

               279         288         297         306         315         324
    TAC AAC AGC AAA ACG AAG AAC AAT GAC TTT GCG ATT GTC ACG GTC AAC AAG GAT
    --- --- --- --- --- --- --- --- --- --- --- --- --- --- --- --- --- ---
    Tyr Asn Ser Lys Thr Lys Asn Asn Asp Phe Ala Ile Val Thr Val Asn Lys Asp

               333         342         351         360         369         378
    ATG GCG ATA GAT GGC AAA ACT ACC AAG ATT ATT ACC CTG GCT AAA GAA GGT TCG
    --- --- --- --- --- --- --- --- --- --- --- --- --- --- --- --- --- ---
    Met Ala Ile Asp Gly Lys Thr Thr Lys Ile Ile Thr Leu Ala Lys Glu Gly Ser

               387         396         405         414         423         432
    TCT GTA CCG GAT AAA ACC AAA CTG CTG GTT AGT GGA TGG GGA GCA ACC TCC GAA
    --- --- --- --- --- --- --- --- --- --- --- --- --- --- --- --- --- ---
    Ser Val Pro Asp Lys Thr Lys Leu Leu Val Ser Gly Trp Gly Ala Thr Ser Glu

               441         450         459         468         477         486
    GGA GGC AGT AGC AGT ACC ACG TTA CGT GCC GTA CAC GTG CAA GCG CAT AGC GAT
    --- --- --- --- --- --- --- --- --- --- --- --- --- --- --- --- --- ---
    Gly Gly Ser Ser Ser Thr Thr Leu Arg Ala Val His Val Gln Ala His Ser Asp

               495         504         513         522         531         540
    GAT GAG TGC AAG AAA TAC TTT CGC TCT CTG ACC AGC AAC ATG TTC TGC GCA GGT
    --- --- --- --- --- --- --- --- --- --- --- --- --- --- --- --- --- ---
    Asp Glu Cys Lys Lys Tyr Phe Arg Ser Leu Thr Ser Asn Met Phe Cys Ala Gly

               549         558         567         576         585         594
    CCA CCG GAA GGT GGC AAA GAC TCG TGT CAA GGC GAT TCA GGT GGT CCT GCC GTG
    --- --- --- --- --- --- --- --- --- --- --- --- --- --- --- --- --- ---
    Pro Pro Glu Gly Gly Lys Asp Ser Cys Gln Gly Asp Ser Gly Gly Pro Ala Val

               603         612         621         630         639         648
    AAA GGC AAT GTG CAG TTA GGC GTC GTG TCG TTT GGT GTT GGG TGT GCG CGG AAA
    --- --- --- --- --- --- --- --- --- --- --- --- --- --- --- --- --- ---
    Lys Gly Asn Val Gln Leu Gly Val Val Ser Phe Gly Val Gly Cys Ala Arg Lys

               657         666         675         684         693         702
    AAC AAT CCG GGC ATT TAT GCG AAA GTG TCT GCA GCC GCA AAA TGG ATC AAA AGC
    --- --- --- --- --- --- --- --- --- --- --- --- --- --- --- --- --- ---
    Asn Asn Pro Gly Ile Tyr Ala Lys Val Ser Ala Ala Ala Lys Trp Ile Lys Ser

               711     3'
    ACA GCC GGG TTG
    --- --- --- ---
    Thr Ala Gly Leu
```

Supplemental Figure S4. The cDNA sequence of prococoonase (proCCN).
